# The project, the everyday, and reflexivity in sociotechnical agri-food assemblages: proposing a conceptual model of digitalisation

**DOI:** 10.1007/s10460-022-10385-4

**Published:** 2022-11-18

**Authors:** Jérémie Forney, Angga Dwiartama

**Affiliations:** 1 Anthropology Institute, Université de Neuchâtel, Rue de Saint-Nicolas 4, 2000 Neuchâtel, Switzerland; 2 The School of Life Sciences and Technology, Institut Teknologi Bandung, Jl. Ganesha 10, Bandung, 40132 Indonesia

**Keywords:** Digitalisation, Assemblage thinking, Socio-technical imaginaries (STI), Switzerland, Indonesia

## Abstract

Digital technologies have opened up new perspectives in thinking about the future of food and farming. Not only do these new technologies promise to revolutionise our way of meeting global food demand, they do so by boldly claiming that they can reduce their environmental impacts. However, they also have the potential to transform the organisation of agri-food systems more fundamentally. Drawing on assemblage theory, we propose a conceptual model of digitalisation organised around three facets: digitalisation as a project; “everyday digitalisation”; and reflexive digitalisation. These facets reflect different relations between concrete practices and representations, imaginaries, and narratives, while representing different modes of agency: the collective, the distributed, and the individual, which, we argue, highlight contrasting ways for human and non-human actors to engage with digitalisation. With this model anchored in assemblage theory, we offer a tool for critically and comprehensively engaging with the complexity and multiplicity of digitalisation as a sociotechnical process. We then apply our theoretical framework to two ethnographic studies, one explores the growth of digital technologies in Switzerland as a way to govern and monitor national agriculture, the other focuses on Indonesia, where small digital startups have begun to dot the landscape. By identifying the material and semiotic processes occurring in each case, we notice similar issues being raised in terms of how digitalisation is co-constructed in society.

## Introduction

The agrifood sector remains critical for livelihoods and employment… Achieving the UN Sustainable Development Goal of a ‘world with zero hunger’ by 2030 will require more productive, efficient, sustainable, inclusive, transparent and resilient food systems… Digital innovations and technologies may be part of the solution. (Trendov et al. 2019:1; FAO Briefing Paper)

Digitalisation in agriculture has gained traction over the past few years and become a buzzword for agricultural development programmes and visions worldwide. References to it are made in regional development plans (e.g. the EU’s Internet of Food and Farms 2020; https://www.iof2020.eu/), to international organisations’ reports (FAO Briefing Paper; Trendov et al. 2019) and intergovernmental summits (De Clercq et al. 2018). The use of the term is widely, and often implicitly, related to a specific vision of what the future of farming and agriculture should look like. Precision farming, automation, and robotics are painted both as an answer to food-security challenges by boosting farm productivity, notably in developing countries (Protopop and Shanoyan 2016), and as a major contribution to the environmental sustainability of agricultural production (Klerkx et al. 2019). Blockchain technologies and big data are shaping hopes for better transparency and traceability in complex value chains (Antonucci et al. 2019). In addition, actors of agri-food systems build diverse imaginaries about the promises of digitalisation, which contribute to their engagement (or non-engagement) with digital technologies.

Leaving aside any discussions over the truth or accuracy of these promises, the fact is that the narratives, around them above all, speak of a desirable future that some powerful actors want to see realised, rather than document today’s reality of the spread of digital technologies in concrete agricultural contexts. At the same time, digital technologies actually contribute to the continuous transformation of agri-food systems, often in ways that differ considerably from those narratives. For instance, the development of a big-data value chain in relation to precision farming in the US has dramatically increased and changed competition between farm input firms, notably by redefining industry boundaries and introducing new actors (Pham and Stack 2018). Imaginaries endorsing the inevitability of the automation of farms help to render the politics of technologies and their impact on labour and rural communities invisible (Carolan 2020). However, they also reconfigure networks, redefine competencies and knowledge and redistribute roles, and transform innovation processes. This has given rise to new ways of thinking about their place in the world and how they reconfigure it (Lioutas and Charatsari 2021). In other words, digitalisation changes the nature of interactions between actors, introduces new actors into existing sociotechnical networks, reconfigures power relations, and creates new alliances and divisions. Many actors confronted with digitalisation processes develop a critical stance that influences the way they engage with various technologies and their proponents (e.g. Regan 2019; Jakku et al. 2019). In this sense, digitalisation in agri-food systems is a very complex and messy set of processes. Faced with this complexity, the risk for research is to focus on the most obvious or dramatic aspects of digitalisation and, as a consequence, overlook other dimensions such as the social changes brought about by the introduction of digital technologies in agri-food systems.

This paper seeks to theoretically structure the messiness of the sociotechnical processes the authors were confronted while conducting research on agri-food digitalisation in Switzerland and Indonesia. We do so, firstly, by reframing digitalisation using an assemblage approach, inspired by the seminal work of Deleuze and Guattari (1988). Assemblage thinking offers three conceptual approaches that are relevant to the interpretation of our encounter with the messiness of digitalisation: the articulation of materiality and narratives; the nuanced understanding of agency; and the attention paid to forces of stabilisation and destabilisation.

Building on these three aspects of an assemblage and addressing them in relation to sociotechnical processes and agri-food systems, the paper elaborates a conceptual model that can be used to analyse various facets of digitalisation (and the ways in which digitalisation emerges as an ordering process). The first facet concerns digitalisation as a project, which refers to its discursive and imaginative dimensions and considers the many narratives that actors develop and engage with in seeing digital tools as desirable solutions. The second facet concerns the messy entanglement of human and non-human actors (machines, ideas, and technologies) in everyday life from which everyday digitalisation emerges. The third facet concerns reflexive digitalisation, which refers to the critical capacity that actors display in their individual and collective engagement with these technologies.

In the second part of the paper, we apply this conceptual model to our case studies of digitalisation processes in Switzerland and Indonesia. The strong contrast between the two countries’ agri-food landscapes in terms of their geography, economy, and social structures allows us to identify very different enactments of digital technologies, related to different projects, the everyday, and reflexive processes.

## Digitalisation as an assemblage

Drawing on the seminal work of Deleuze and Guattari (1988), assemblage thinking has inspired social-science scholars in several fields and disciplines, notably urban-, development-, and health studies (e.g. McFarlane 2011; Li 2007; Duff 2014). Agri-food scholars have only recently started to engage with this framework and its concepts, particularly those studying regional bioeconomies (Lewis et al. 2013), metrologies (Rosin et al. 2017), agri-environmental governance (Forney et al. 2018), global value chains (Jones et al. 2019) and reconfiguration processes instigated by agri-environmental organisations (Bentia 2021). Here, we expand this field by applying assemblage thinking to the study of agri-food digitalisation as a complex social phenomenon.

Deleuze, in a discussion with Parnet, defines an assemblage as “a multiplicity which is made up of many heterogeneous terms and which establishes liaisons, relations between them across ages, sexes and reigns—different natures”. He adds that the unity of the assemblage results from “co-functioning: it is a symbiosis, a ‘sympathy’” (Deleuze and Parnet 2002, 69 [1977]). In other words, assemblages are entities made up of elements that are heterogeneous in nature, where the making and unmaking of relations between these elements essential to the dynamism of the assemblage. Digitalisation can usefully be framed as a multiplicity made up of changing relations between heterogeneous elements. Digital agricultural technologies reassemble, in new ways, long-standing relations between farmers, fields, machinery, animals, advisors, science, markets—to name but a few—and introduce new elements into the system: databases, algorithms, technicians, etc. This rather descriptive use of the concept of assemblage can be expanded by considering the emergent nature of assemblages. Assemblages “are not defined by their components”, the things they bring together. Rather, they are defined by “what they produce” (Buchanan 2021: 47). What counts then are the outcomes of this coming together of things. However, the real analytical power of assemblage, as a concept, cannot be reduced to these broad definitions.

In this section, we want to dig deeper into assemblage theory and develop three aspects of assemblages that characterise these complex conceptual constructs. *First,* Deleuze and Guatarri locate assemblages at the interface of two planes of existence, two interrelated dimensions: “the machinic assemblage of bodies (content) and the collective assemblage of enunciation” (Buchanan 2021: 33). A machinic assemblage is configured through elements that are physically bound and held together, whereas an assemblage of enunciation is an expression of meanings and narratives that are formed between humans, ideas, and technological non-humans (Dwiartama and Piatti 2016). Understanding the entanglement of material (non-discursive) and semiotic (discursive) relations is the first step in grasping the complexity of the concept of digitalisation as an assemblage more fully.

*Second,* the notion of distributed agency as developed by Bennett (2010) in her attempt to better integrate objects into political theory points to the fact that agency always emerges from assemblages of humans and non-humans. As McFarlane (2011) puts it, agency in an assemblage “both forms a coalition and yet preserves something of the agency or impetus of each element” (566). Consequently, no singular element of an assemblage can control its outcomes. Each element always depends on others to develop its actions and is inevitably influenced and mobilised by others. This, however, also implies that each element does have this fundamental capacity to influence others and, in doing so, the broader assemblage. Agency here is related to the concept of desire, which takes a central place in Deleuze and Guattarri’s work. Desire expresses the will of elements to make the assemblage cohere, or to escape relations in order to assemble elsewhere. Desire is present from the beginning, while agency can be seen as the translation of desire into the capacity to act.

Third, the fact that elements of assemblages are sometimes drawn together and attracted by other assemblages points to the fragile nature of assemblages. Assemblages are always in the making and subject to constant reconfiguration. The concept of ‘line of flight’ crystallises the propensity of assemblages to experience destabilising forces that attract elements and “carry them away” (Deleuze and Guattari 1988: 510). Referring to Deleuze and Guattari (1988), Dewsbury (2011) distinguishes, but also conflates, these two ideas: lines of flight here refer to the nature of entities and assemblages to deconstruct a structure and disrupt a process of formation, while lines of articulation refer to the tendency of these very entities to simultaneously construct and reinforce an assemblage’s formation. When illustrating the dynamics of local food initiatives, for instance, Dwiartama and Piatti (2016) show that these initiatives are held together by a process of always becoming, where actors continually engage with articulation and flight, creating a vibrant and transient, but at the same time stable and sedentary, assemblage. The processes at play do not necessarily lead to one element staying or leaving—and it should not be seen as such. Lines of articulation might be multiple and compete with one another to define not only if, but also how elements will assemble. Similarly, diverse lines of flight might pull elements in different directions.

In this paper, we look at digitalisation in agriculture as a relatively new reality brought into being through the introduction of new technological assemblages that combine a range of lines of flight and articulation. In that way, they contribute to transforming existing agri-food assemblages.

## The three facets of digitalisation

The intellectual journey leading to this paper started with intense discussions between the two authors while comparing their ongoing research on digitalisation processes from the perspective of the “everyday”. At the time, both authors collaborated in a project on agri-environmental governance from an everyday and assemblage perspective (Forney 2016; Forney et al. 2018). By “everyday” we first understood the mundane and ordinary aspects of digitalisation, often overshadowed by “spotlight digitalisation” (see the introduction to this symposium). We felt the need to better articulate the tensions and complex relations between what we observed during our fieldwork and the grand narratives that crowded the media and the internet on “digitalisation”. We then began a long-standing engagement with this messiness of digitalisation in the context of agri-food systems, raising new questions and working with new concepts. This resulted in the conceptual model elaborated here, which articulates digitalisation as a project, as the everyday, and its reflexive aspects.

### Digitalisation as a project: the realm of sociotechnical imaginaries

Many narratives have been developed of desirable digital futures in agriculture and food (see e.g., Adams et al. 2009), providing digital answers to what seem to be urgent problems that need to be addressed. Those narratives point to the first facet of digitalisation today, which we refer to as *digitalisation as a project*. Here, we consider mainly the discursive and imaginative dimensions of digitalisation as a project. The vision of digitalisation as the future of agriculture seems to be gaining in strength and is shared by a growing number of actors. It underpins countless research and innovation programmes, political agendas, and attracts considerable financial investment aimed at bringing this future about.

Jasanoff and Kim (2009) have advanced the powerful concept of ‘sociotechnical imaginaries’ (STIs) to analyse idealistic and normative visions of sciences and technologies and their ordering effects. Jasanoff defines STIs as “collectively held, institutionally stabilised, and publicly performed visions of desirable futures, animated by shared understandings of forms of social life and social order attainable through, and supportive of, advances in science and technology” (Jasanoff 2015: 84). The term “desirable futures” is important here as it highlights the moral and political dimensions of STIs, which goes far beyond mere technology and science. STIs also encompass “social imaginaries” that encode “collective visions of the good society” (Jasanoff and Kim 2009: 123).

We adopt the concept of STIs here to acknowledge both the power of imaginaries that become dominant at a large scale and the inevitable discrepancies that occur when they are realised locally. However, while Jasanoff and Kim’s STIs focus largely on shared, stabilised, national imaginaries,, concerning digitalisation there are still many competing alternative visions, or “space[s] of disagreement”, about what the digital future of agriculture is and should be (Carolan 2019). We therefore propose the concept of a sociotechnical “project” as an alternative to highlight this coexistence of many projects and their evolving nature.

As we see it, the projective dimension of digitalisation lies along an axis that links up more stabilised and widespread imaginaries on the one hand with more flexible and localised visions of desirable futures on the other hand, as opposed to Jasanoff and Kim’s imaginaries that refer to rather well-defined, dominant, projects. In this sense, Le Velly’s (2018) deconstruction of the French word “projet” gives us a more flexible framing for STIs. Drawing on the work of Bréchet et al. (2009) who define a project as “a fuzzy operative expectation of a desired future” (Bréchet et al. 2009, p. 41), Le Velly elaborates that:

The project is an operative by means of which individuals imagine a future that they deem desirable and conceive of its broad characteristics… To be able to act together, people must have common landmarks that give meaning and direction to the creation and evolution of the collective.

Le Velly also distinguishes between ‘the project’ and the ‘setting of rules’, on the one hand, and the ‘implementation of the project’, on the other hand. This flexible approach allows him to identify partial disagreements and divergences. For him, the notion of “project” does not imply its full acceptance by all actors. Rather, the project has “the ability to unite actors whose aims are not identical”. Hence Le Velly emphasises the importance of the notion of a “desirable future” that binds the project to (collective) agency.

### Everyday digitalisation: the multiplicity of digital assemblages

In a perspective where the coproduction of digitalisation assemblages combines expressions and content (or enunciations and machines), imaginaries have permeated the very materiality of agriculture—the ways farms are organised socially and physically. Many actors contribute to the shaping of new technologies upon their introduction into new assemblages. In fact, the actual practices that illustrate the penetration of digital technologies into localised agricultural- and food systems seldom match the big narratives about digital agricultural futures and what they should look like. While the project projects their ideal future, digitalisation in everyday practice has to deal with obstacles and compromises that make up the social lives of technologies. As noted in regard to STIs, there are pre-existing imaginaries that shape the way digitalisation is interpreted in a specific context.

Just as an everyday governance perspective focuses on the translation and appropriation of governance instruments in everyday life, researching *everyday digitalisation* forces us to look beyond techno-utopian narratives and to understand change in less linear ways. When considering other social science analyses of of digital tools and their everyday use, e.g. computing and media consumption (Haddon 2006), smart grid (Nyborg 2015), and technology adoption/transfer (de Laet and Mol 2000), it becomes obvious that these innovative technologies are used by actors on the ground in ways that deviate from their original purpose. Sometimes they are not even used at all. Focusing on the integration of new technologies at the everyday-life level and on the active role that users play as innovators, we find echoes of insights from domestication theory at the micro level (Haddon 2011). However, the everyday is not limited to the micro level of intimate relations between actors and technologies. As highlighted by Furlong et al. (2019), the idea of the everyday is made up of interplays between macro social relations and structures, and micro practices and daily tactics.

From the flattened perspective of assemblage thinking (Deleuze and Guattari 1988), macro and micro levels merge through the relations that constitute the assemblage. In the making of the everyday digitalisation of agriculture, the “domestication” of new technologies has to be understood as a continuous process that starts at the very beginning of the innovation process, in the making of the technology itself, and follows a long chain of relations and processes of translation and mutual adjustments as the technology enters existing assemblages at different scales (from a global food chain to the farm). In other words, while digitalisation is centred on the representational and expressive dimensions of the assemblage, everyday digitalisation refers to its machinic dimension. In this sense, everyday digitalisation points to the lived experience of digitalisation of many actors as opposed to the dominant visions of digitalisation as a project. However, both digitalisation as a project and everyday digitalisation insufficiently illustrate the nature of digitalisation as an assemblage and the different forms of agency involved in its achievement. We also need to consider the role played in this process by reflexive digitalisation.

### Reflexive digitalisation: or digitalisation as an object of critical enquiry

The notion of reflexivity, as a critical assessment of the impact of technologies and an expression of agency, plays an important part in understanding digitalisation. This concept is central to the reflexive-society paradigm proposed by Giddens (1984). It relates to the fact that, for new technologies, the demise of the optimistic and positive conception of progress that characterised late modernity obviously had a major impact. In social theory, reflexivity has often been related to agency. The capacity to think critically about one’s own actions and their context has been presented as a condition for being able to act consciously and autonomously (e.g. Chernilo 2016). Interestingly for our purpose is Chernilo’s articulation of reflexivity and its relation to the projective dimension of human desire: “Reflexivity is fundamentally exploratory and transforms, reorients and prioritises our personal concerns into the projects that we seek to realise in society” (Chernilo 2016: 198). However, as Martin (2006: 255) puts it in her study on gender relations at work, reflexivity is not systematic. While non-reflexivity is a central concept for understanding “undesired” consequences, reflexivity refers to the cognitive activity that casts a critical look at both the visions guiding projects and the effects of the deployment of technologies in the everyday. Littler (2005) proposes the concept of relational reflexivity to overcome some limitations of an individualistic understanding of reflexivity. Drawing on Haraway’s notion of diffraction, Littler grounds reflexivity in the relations between multiple perspectives, as opposed to a narcissistic reflexivity that will always be limited by a unique positionality.

Here, we understand reflexive digitalisation as a collective capacity and practice that emerges from a critical engagement with digitalisation, both as a project and everyday practice, and from an openness to the perspectives of others. Changes introduced by digitalisation rearticulate assemblages and provoke reflexive adjustments and repositioning, both in the machinic and enunciative dimensions. This partly resonates with Orlikowski’s (1992) work on the duality of technology, in which a corresponding interaction between technology and its users enables them to reflect on their desires, intentions, and norms.

Reflexive digitalisation enables actors to redefine their relations with technologies, actors, crops, animals, ideas, and objects, as well as their plans and objectives. While acknowledging the necessity of critical counternarratives, we conceptualise reflexivity here not as being related to objectivity or notions of authenticity and truth. Reflexive digitalisation is understood here as complementary to the project and the everyday. The concept guards, we argue, against the temptation to resort to binaries that the first two concepts outlined above might inadvertently encourage: discourse/practice; abstract/concrete, etc.

Following Lash’s (2005: 18) comment on reflexivity in relation to “technological culture”, reflexive digitalisation is to think, to do, and to communicate, all at once. Reflexivity should not be understood as external reflection from afar. On the contrary, reflexivity is internal to digitalisation, which means that doubts, questions, and critical assessment continuously partake in the construction of digitalisation.

### A conceptual model of digitalisation

**Fig. 1:**
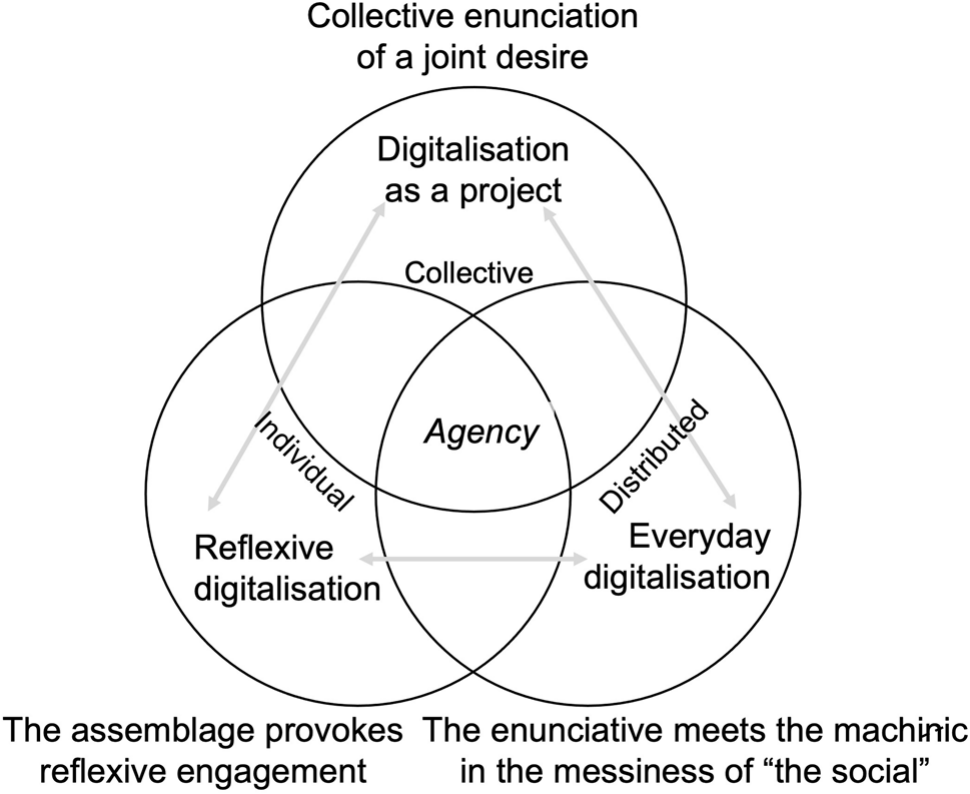
A conceptual model of the three facets of digitalisation

Below, we construct a conceptual model of digitalisation based on these three facets or dimensions of digitalisation—the project, the everyday, and reflexive digitalisation (see Fig. 1). We argue that where digitalisation occurs in agriculture, there is an interplay between these three facets. This interplay does not resemble a cyclical process and the phases do not occur in succession. Rather, they take the form of a multiplicity where they dovetail together and occur simultaneously. Of course, it is logical to think that a desire or vision would consequently be followed by its encounter with the everyday, to be considered reflexively by actors later on. However, our research showed that within the everyday, there was a continuous endeavour to coproduce visions of digitalisation, or to critically question its very existence—each contributing to a comprehensive idea of digitalisation itself. It is perhaps safe to say that in a digitalisation assemblage, there is intentionality and unpredictability, stability and transience, discourse, practice, and abstraction—all occurring at the same time.

Simultaneity notwithstanding, rather than picturing these three facets in a single frame, our model pays attention to dynamics—the ways in which each facet informs, provokes, and transforms the others, as illustrated in Fig. 1. Digitalisation as a project concerns how a particular type of enunciation is coproduced and expresses a joint desire. This desire is collective, in the sense that although individual actors can have desires too, the nascence of a project is always the result of a collective endeavour—either in terms of how the idea comes into being or how it is communicated, coordinated, and translated into strategies and actions, involving some form of reflexivity and encounter of the everyday (no matter how universal the process is, see e.g. Enticott 2012). This is counterintuitive to Le Velly’s (2009) view that a project can be individual or collective. We argue that no project can manifest itself through individual agency alone, and understanding the collective agency of a project, therefore, becomes pertinent in depicting digitalisation.

Assemblages always combine the enunciative and the machinic. We argue that this is how the everyday manifests itself in the messiness of the social (which results from complex interactions between humans and non-humans). Here, elements and actors mingle and translate the digital imaginaries into everyday encounters and tactics. Actors negotiate within the assemblage to maintain a certain stability (what Deleuze and Guattari refer to as lines of articulation), while others resist, contest, and engage in lines of flight, creating a precarious assemblage that is always in the making. Actors often realise that everyday practice is not as ideal as they envisioned it to be; but through these messy relations, desire is also coproduced. The centrality of distributed agency needs to be re-emphasised here. It is a kind of agency where human and non-human actors equally reconfigure relations and assemblages, bringing new ones into being.

While the interplay between the enunciative and the machinic within assemblages has often been discussed in the literature (Deleuze and Guattari 1988; Dewsbury 2011; Dwiartama and Piatti 2016), as well as how reflexivity is an integral, albeit counterintuitive, part of dominant narratives of a project, we suggest that reflexivity is also an important dimension of the everyday. Digitalisation as a project or the everyday (or a combination of both) more often than not provokes actors to engage reflexively with the assemblage. Experience of the machinic—its incompatibility, recalcitrance, or messiness—might transform and coproduce new imaginaries, but it can also translate into everyday tactics and actions. In the next section, we seek to test this conceptual model through case studies of digitalisation projects in Switzerland and Indonesia.

## Case study reflections: agri-food digitalisation in Switzerland and Indonesia

Digital technologies are diffusing globally, based on standardised languages and tools. However, these technologies are just potentialities that are enacted in various ways in localised assemblages. This enactment of technologies follows diverse processes as digital tools enter specific assemblages. As shown in our conceptual model, they give birth to diverse projects, everyday realities, and reflexive processes. This means that the story of digitalisation is not a story of diffusion (of digital agriculture), but a story of how digital technologies enter existing assemblages, are shaped by them, and in turn transform them.

Indeed, rather than producing a uniform and globalised reality of digitalisation in agriculture, these transformations and translations create multiple “global assemblages”, in Ong and Collier’s (2005) sense, where global processes result in very specific, localised, realities. Those localised assemblages are shaped by interactions between technologies as potentialities, localised politics, and materialities. Below, we present two case studies—one from Switzerland, the other one from Indonesia—which illustrate this diversity of enactments of global digitalisation processes in agriculture in specific locations. We examine how the specificities of localised agri-food assemblages contribute to the shaping of diverging digitalisation projects, the production of particular everyday uses of digital technologies, as well as engendering context-specific reflexive engagements with particular processes of digitalisation.

### Data collection methods

This article emerged from a discussion between the authors in which they tried to make connections between their separate case studies. These were conducted with relative autonomy within a shared project on everyday digitalisation in agri-environmental governance. In this sense, this article builds on an approach in terms of the everyday applied to two case studies that reflect very different aspects and configurations of digitalisation processes in agri-food systems. As a consequence, the objective of this article is not so much to give a detailed account of the research results of our case studies, but rather to offer an illustration for the conceptual model through the case studies.

The data used in the Swiss case study resulted from an ethnographic approach that combined direct observation at several large events related to the digitalisation of agriculture in the country, and a series of semi-structured interviews with multiple actors involved in the governance of agriculture. Our sample was constructed by actively seeking stakeholders—through online research, notices in the press, and personal recommendations—who were directly or indirectly involved in digitalisation processes. Some of these people are drivers of this transition, others are people whose activities are affected by it. This allowed us to obtain a relevant sample of 23 interviewees, including people active in public administration (5), agricultural organisations (4), agricultural training and advising (5), certification (2), agri-digital projects (4), and farming (3).

Likewise, the Indonesian case study employed a combination of interview-based ethnographic study, desk (and digital) evaluations, group discussions, workshops, and participant observation. There are three categories of actors that were engaged in the interview process: (1) actors in a digital startup, (2) the stakeholders involved with the startup, and (3) state authorities and other non-economic actors. In regard to the farmers, we interviewed some that were associated with the startup, as well as independent farmers to whom we had access through state agencies. In total, we conducted 20 in-depth interviews, including with startup owners and staff (4), farmers (8), government officials (5), and members of the financial sector (3).

In both case studies, our analysis follows an approach that aims to test the conceptual model through empirical illustrations. Notes from observations and interviews were coded using categories that emerged from the data itself as we sought to compare the actors’ perspectives on current processes and challenges related to digitalisation. Data analyses from both cases were brought together, combined, and synthesised through a series of discussions and workshops. As we mentioned earlier in this paper, our analyses of the empirical data were based on the conceptual model that we co-constructed during the course of our joint collaboration, regardless of the different, and often contrasting, nature of our case studies.

### Switzerland and the rise of digital bureaucratisation

Switzerland’s physical geography is characterised by two mountainous areas and a plateau in between. On the one hand, the Alps and Jura ranges cover one third of the nations’ territory, which historically has given rise to the development of dairy farming and cheese production. On the other hand, the plateau between them forms a large plain of arable land, where diverse agricultural activities take place amidst a high population density. Because of these geographical characteristics, and due to a strong protectionist agricultural policy in the twentieth century, farms in Switzerland have remained comparatively small.^1^ The majority of farms in Switzerland are family farms, which are very often specialised, well equipped with machinery, and embedded in a dense network of professional organisations.

Swiss agriculture, furthermore, is also characterized by the strong influence that the federal state has on agricultural policy, yielded through the direct farm payment system. Since the 1990s, farmers have received public incentives for their contribution to the delivery of environmental and social services as part of achieving a multifunctional agriculture (for details, see Forney 2016; Mann 2003). In practice, the regional administrations are in charge of collecting and verifying farm data. At the same time, third-party certification schemes and food labels have flourished in food retail here. The conjunction of the direct payment system and multiple certification schemes have heightened the need for accountability and traceability in agri-food assemblages (Forney 2021). As a consequence, bureaucratic monitoring processes—produced by configurations of human (agents of the administration, controllers, certifiers, etc.) and non-humans (forms, procedures, databases, etc.) actors—have become essential in making agriculture viable politically and economically (ibid.). Both agricultural policies and markets are dominated by an audit culture, where the gathering of data about farms and farming activities has become essential. For many farmers, bureaucratic controls, activities, and paperwork linked to accountability are becoming an overwhelming burden.

Digitalisation projects have been generally presented as a solution for most of the problems and challenges encountered by Swiss agriculture, including reducing the use of chemicals, more efficient traceability systems, and more transparent supply chains. However, following recent studies (Groher et al. 2020), Swiss farmers’ interest in digital farm technologies seems to be limited. The comparatively small size of Swiss farms, as well as incongruities between technical solutions and local needs, have proven to be an obstacle to the diffusion of smart farming technologies.

Since the end of the 1990s, the production and collection by regional administrations of agricultural data and statistics, notably in relation to agri-environmental policies, has been progressively digitised.^2^ At the same time, many small companies have emerged, offering digital tools to help farmers translate their practices into data required by the administration. In the early 2000s, the Federal Office for Agriculture (FOAG) became directly involved in the digital governance of agriculture, offering political and financial support for specific digital tools and creating its own databases. These developments and the introduction of new technological—non-human—elements have resulted in a more centralised governance of digital agricultural bureaucracies. However, organising the latter remains messy and fragmented as regional and sectoral systems continue to develop in parallel. Solving the issues of both the overload of administrative work for farmers and the fragmentation of the digital data landscape has progressively become a central aim for the governance of agriculture in Switzerland.

### Indonesia, startups, and the privatisation of the agri-digital space

Agri-digital governance in Indonesia arguably offers a rather peculiar case in relation to its assemblage. On the one hand, with more than 30 per cent of its land being dedicated to agriculture, the sector contributes 13 per cent of the country’s GDP and employs 34 per cent of the labour force (National Statistical Bureau, 2019). The majority of farms are managed as small holds, with more than 14 million farm households owning less than 0.5 hectares of farmland. On the other hand, Indonesia is becoming more urbanised and more than 50 per cent of its population now lives in urban areas, particularly the metropolitan cities of the island of Java. On Java alone, the rise of the use of the internet has been astonishing, with more than 80 million people, or more than 58 per cent of the island’s population, being active internet users (JakartaGlobe, February 2018).

In line with this digital literacy, the growth of digitalisation in Indonesia in general has been extraordinary. The Indonesia Venture Capital Outlook 2017 Report, a joint Google—A.T. Kearney initiative which examined trends of investment in Asia, provides a snapshot of Indonesia where investments in digital startups (mainly fintech and e-commerce) have increased 68 times in the past five years, with a total value of up to USD 3 billion in 2017. A.T. Kearney also estimated that there are more than 2,000 digital startup companies in the country. There are three digital giant tech companies in Indonesia that are tapping into this growing market: Go-Jek (a local Uber-like online transport service using mainly motorcycles; USD 1.8 billion worth of investment), Tokopedia (e-commerce; USD 1.4 billion), and Traveloka (online travel agent; USD 500 million). The former two companies are also expanding into the agri-food sector.

It is not surprising then that startups have been at the forefront of the digitalisation of agriculture in the country. The absence of state control, strong support for innovation from private industry, and the involvement of urban youth in the agricultural sector (see e.g. Dwiartama et al. 2016; Dwiartama and Suheri 2016) have opened up ways for agri-digital startups to grow and flourish. Investment trends in startups have also created an assemblage of actors whereby venture capitalists, angel investors, seed funding schemes, and business competition dictate how these agri-digital startups align themselves with the development narrative. This very vibrant process also involves multinational corporations, tech giants, international networks, and, to some extent, the government. In the following section, we will explore some of the growing agri-digital startups that we have engaged with more deeply over the course of our study, while also highlighting the role of government in creating a certain narrative that enables these startups to flourish in the everyday life of rural farmers.

### ***Digitalisation projects: dealing with the messiness of data***

Despite their contrasting national contexts, Switzerland and Indonesia both consider digitalisation as they way forward for dealing with the messiness of agricultural data although they do so in very different ways Below, we detail two digitalisation projects that translate this wider challenge into concrete, more focused, and specific interventions.

As projects, the idea that digital technologies will make the collection, circulation, and processing of agricultural data easier has gained traction among many actors, both in Switzerland and Indonesia. In Switzerland, one dominant digitalisation project has therefore centred on the objective of making flows of data between actors in the agricultural sector smoother in order to make the governance system more efficient. Barto is a Swiss digital farm management that pursues this objective. It emerged in 2015, initiated by two semi-public actors (Identitas SA and Agridea). In 2018, Barto became a private company whose shareholders were all companies or organisations with a stake in agriculture. With the official support of the Federal Office for Agriculture (FOAG), Barto was established as a solution to deal with the messiness of agricultural data at the national level by combining and more clearly articulating certain aspects of digitalisation. The actors behind Barto aims for this tool to become a central platform that farmers can use to upload and share their data with diverse partners within economic or governance networks, or use it as part of diverse farm management tools. The simplification of farmers’ bureaucratic life lies at the core of the narratives put forward by proponents of Barto.

Barto aims at becoming a central node in the network of agricultural digitalisation in Switzerland. While smart farming tools would collect data, the data collected would be automatically integrated into Barto’s database. Farmers could also input administrative data required about their practices and farms by different partners into its database (e.g. farm structure data, livestock data, land data, data about their practices, etc.). This data would then be shares with relevant partners with consent from farmers. In this way, the digitalisation project of Barto through its central platform would rearrange the broad and fragmented assemblage of Swiss digital agriculture.

In Indonesia, agri-digital startups are mostly established upon a certain food utopia which paints a picture of how both technology and Indonesia’s youth drive change in its existing, rather broken, food system. They are portrayed to provide the solutions to achieving food security and sustainability here. The “help farmers, save farming” narrative is present in the objectives and taglines of these startups. Some appeal to the imagery of old traditional farmers needing technological support to stay in business, or peasants that are tied to debts and in need of financial resources, or the inability of small-scale farmers to access the market and receive a better price for their produce. They create their own digitalisation projects, and collectively planned actions to enact digitalisation in the agri-food landscape. Through their narratives, they try to appease not only farmers, but also the general mass of internet users in urban areas who put a particular interest in contributing to a better food system.

One of the prominent agri-digital startups in Indonesia, eFishery, perhaps perfectly illustrates this case. Established in 2013, eFishery was Indonesia’s first startup in the aquaculture sector, offering IoT-based technological solutions and data management support to freshwater fish and shrimp aquaculturalists. eFishery was founded by three alumni of one of the largest technology universities in Indonesia, who found a solution to efficiently feed catfish through an automatic feeding machine they had invented. Their vision was to revolutionise modern aquaculture through technology and business ecosystems. In the words of its CEO, “I believe that eradicating poverty and hunger can be done by disrupting the agriculture sector through technology”.

eFishery’s technology was trialled between 2013 and 2016, and the team participated in a plethora of startup competitions to gain venture capital, including the Tjiputra Group creative business cup, AquaSpark, 500Startups, Ideosource, ICT Award from the Ministry of Communication, USAid’s Tech4Farmer Challenge, Google’s Launchpad Accelerator, and Rotterdam Olympics of startups. This list illustrates the immense assemblage that had meanwhile sprung up in the background. eFishery has huge potential only in regard to its fish feeding technology, but also in regard to its ability to mine farm-level data (size and scale, water quality, harvest periods, fish production, farm expenditures). It also stores the data collected in a cloud database. In 2016, eFishery entered its commercial release phase, being categorised as an A-series startup (receiving USD 1–3 million of funding).

In the two cases described, digitalisation projects are to a certain extent coproduced through the enunciation of a common (or rather, joint) desire. While each digital actor (in this case Barto and Fishery) seeks to bring its own project to fruition, both can be seen as “projects” of a wider assemblages. Note how Barto was established as a joint desire of the federal administration, the private sector, and cooperatives; as a digital startup, eFishery was shaped by the cofounders, but also venture capitalists and business competition platforms from within and outside Indonesia. Projects, in this context, may have arisen from localised imaginaries that gained traction as part of a wider assemblage (as in the case of eFishery) or nationwide desires that are translated to the local scale (as in the case of Barto). Both projects are, to borrow Jasanoff’s (2015) words, collectively held, institutionally stabilised, and publicly performed. For example,, the exposure of startups like eFishery to international competitions and business ventures not only renders them visible to the public, but stabilises them as a path towards an imagined agri-food future that is dependent on digital technologies and creative ideas. For Barto, the official support of the federal government, in addition to the backing of private shareholders, legitimised this digitalisation project.

### Everyday digitalisation: rearticulation of digital agri-food assemblages

The two projects presented above aim to order the messiness of data as that is collected in different contexts, in different ways, and using different approaches. From an assemblage perspective, such ordering can be described as a reordering, a redefinition of the relations that already exist within the assemblage. In this section, we look at what happens when projects interact with the everyday dimension of current assemblages, trying to reconfigure their messiness. As we will see, this process of translation from a projective dimension of digitalisation to the everyday is made through diverse reappropriations and resistances. This results in diverse outcomes that often do not align with the objectives of the project.

To begin to exist in the everyday dimension, Barto’s project had to be translated into actual technology and gain concrete economic stature. The technical reality of Barto is based on the reuse of a smart farming tool, 365FarmNet, which was developed by the German agricultural machinery company Claas. This software is already used in several European countries (Austria, Germany, France, and Poland) and has around 40,000 users. The partnership between Barto and 365FarmNet led to the adaptation of the original software to the Swiss context. A company was established to manage the platform, with two major shareholders each owning one third of the shares: Identitas AG, a semi-private company controlled by the federal state and in charge of monitoring and controlling farm animal circulation at the national level, and FENACO, the largest farmer cooperative in the country, which occupies a dominant position in several agriculture-related sectors thanks to its numerous subsidiaries. While there is no majority shareholder in Barto, the remaining shareholders—farmer organisations and the semi-private extension service Agridea—only own a small proportion of the shares. Interestingly, FENACO has a direct connection with Claas, the German owner of 365FarmNet, through its subsidiary Serco Landtechnik AG, who is the official importer of Claas machinery in Switzerland.

Consequently, Barto as a project resulted from a reconfiguration of existing relations, assigning a key role to formerly marginal actors: Barto and the German company Class (through the 365FarmNet software), where the former became a central node in the new digital assemblage. This reconfiguration met the resistance of existing lines of articulation in the digital farm data assemblage and was challenged by alternative reorganisation processes. First, intricate relations between some of the central partners blurred the simple image of Barto as a neutral platform. FENACO’s engagement in Barto, for example, highlighted the key role played by market competition for farm related data. The central involvement of foreign economic actors (Claas and 365FarmNet) also raised questions about the national sovereignty of data, as data related to public policies would be stored on a foreign server. Second, and more implicitly, the central position that Barto planned to occupy in the circulation of data clashed with the current role and function of regional administrations and their regional databases. These actors provide the interface between farmers and databases in Swiss agricultural policies. The vision offered by Barto of a centralised system of data collection would make this role obsolete. These mid-level bureaucrats, however, also have the role and legitimacy to verify the accuracy of the data collected on farms, role fundamental to generating trust in the accountability of the system related to direct farm payments. This is something that a company like Barto could not achieve.

Maybe as a result of these tensions, the Barto platform, despite being very visible in national discussions about the digitalisation of agriculture, has struggled to enrol actors from the Swiss agri-food sector. This statement has to be nuanced by the fact that the platform is still in its development phase. However, only a minority of farmers have shown an interest in it and have started to use it. Furthermore, only a few external actors have developed a plug-in module and to date, the platform only offers 14 modules. Four of these modules were developed by Barto for the collection of animal and farm data required by Swiss agricultural policy, and seven were imported from the original software 365FarmNet.

In enacting their projects in the everyday, eFishery have worked with, and had their technology used by, hundreds or even thousands of farmers. They collaborated with the government to access funding, markets, networks, and policy support. Under the banner of Agriculture 4.0 and smart farming, for instance, the West Java provincial government endorsed eFishery and opened ways for their technology to be adopted by aquaculture farmers in many regions in the province. The “digital fishery village” programme, for instance, was enacted through the involvement of eFishery and BRI, an Indonesian state-owned rural bank, in order to establish a basis for farm data consolidation.

This reconfiguration translates to ways in which farmers experience the everyday. Some of the aquaculture farmers affiliated with eFishery mentioned that they had seen the direct impact of the technology on their production costs and yields. Some of the younger generations of farmers were quite enthusiastic about the technology and its potential. There is an observable change in the way farmers farm when they incorporate the technology into their daily routines. As such, the materiality of the non-human actors played a relevant role in their everyday practice. One farmer described his experience of using eFishery’s automatic feeding machine, which was designed to be controlled remotely. He was curious about the device and decided to keep an eye on it to see how it worked. Instead of closely observing the fishponds, the farmer now closely observed the device that observes the fishponds—which defeats the purpose of automation and remote monitoring, at least in the short term. These everyday challenges are ubiquitous and require some form of sociotechnical adaptation, tweaks, and fine-tuning.

Some of these lines of articulation, however, are met with recalcitrance. A few farmers were dismissive of the idea that disruptive technology would improve their production. The regarded this new technology with scepticism because it pushes them out of their comfort zone. Some were worried that the cost of renting eFishery’s automatic feeding machines and the energy cost associated with them would further increase their already rising production costs. The majority of farmers only have one to three fishponds, and using eFishery would not bring them significant benefits compared to the costs and hassle of shifting to IoT.

What is even more fundamental is the fact that digitalisation projects, as any other projects, tend to disrupt and reconfigure existing assemblages. To illustrate this, eFishery started off as a technology provider for fish farmers. The technology captures real-time water-quality data and responds to this by feeding fish accordingly. By registering at what stage in the feeding cycle the water quality decreases, the technology enables the efficient use of fish feed, thus saving farmers money. Another technology measures the size of the fish, informing farmers when to harvest. These machinic relations between the human and non-human actors have wider consequences for the agri-food landscape and those involved in it, such as fish-feed companies, markets, consumers, and financial institutions. Through its cloud-connected platform, eFishery can capitalise on farm-level data, linking this up with feed companies that sell their products to farmers. eFishery is able to exclude middlemen as it connects farmers directly to restaurants and retailers by informing the latter about which farms are about to harvest.

### Reflexive digital assemblages

Translating projects into everyday digitalisation practices leads to a series of transformations in the way that actors conceive of their role and identity as farmers, as bureaucrats, as digital technicians, etc. In other words, the encounter between projects and the everyday is associated with reflexive processes about actors’s role in the assemblage. Below, we provide some examples of reflexive digitalisation in Switzerland and Indonesia.

Barto’s project is criticised by some for being disconnected from the reality of farmers. This would partly explain why Barto has found it difficult to take off. As explained by an executive from a farmer organisation:

Barto is an integrated system, which, from our point of view… does not meet the expectations of the majority of Swiss farmers. It may meet the needs of an elite of farmers who are connected at every level. But the farmer from Appenzell who has 13 hectares on average, I don’t know if he will have a robot at every level…

However, issues of scale are not the only problem identified with Barto. Concerns over data ownership and control are expressed by many. The complex set of relations that characterises collaborations around Barto, including German companies, the large cooperative FENACO, and the federal state raise questions about hidden agendas and the diverse interests behind the official objective of easing farmers’ bureaucratic burden with digital and smart farming technologies. Speculations over the economic interests of private actors join fears of increasing state control over farmers’ activities. Indeed, the public–private partnership at the core of Barto’s project seems to increase two risks associated with digital data: the commercial exploitation of farmers’ data and digital surveillance. Commenting spontaneously on Barto, a farmer we met because he used some smart farming technologies (mostly connected to tractors and machinery) summed up these concerns:

I don’t want my data to be used by others! Because I see it coming. FENACO, they have this Barto project… all this is like loyalty cards… digitalisation should help me in my work, but it should not be a means to sell me stuff I do not need. That’s a bit of what it is. We know well what happens with all this data! (CAC, farmer)

They will try to use the data for commercial purposes and then, on the other hand, also spy on us. The FOAG, they will be able to say: you’ve put this stuff two centimetres too far… And then this is no help anymore, it becomes policing! (CAC, farmer)

Farmers’ distrust on other actors in the everyday materialisation of Barto’s project also related to the desire to avoid systemic dependency, as suggested by the executive of a regional farmer union:

But you feel that maybe, if you work with FENACO, you’ll need to have a Claas [tractor] and all the stuff that goes with it… and then, you don’t have a choice anymore. By reducing choice, farmers see they lose some freedom and autonomy. (YH, farmer union)

From the perspective of Swiss farmers, reflexive digitalisation points essentially to the risk of a weakening of farmers’ position in the digital assemblage. But farmers are not the only ones critical of Barto. The creator of one of the regional databases stated, in a dispassionate way, that Barto wanted to build a “data monopoly” and become a key actor in the digitalisation of Swiss agriculture. This highlights the strategic position that the platform aims to occupy in the streaming of agricultural data. As mentioned earlier, the regional administrations currently manage the agricultural databases related to the direct payment system. These actors tend to resist the idea of a private actor centralising data collection and coordination. As noted by a representative of one of Barto’s minority shareholders, letting go of the former articulation of data flows is not easy:

People want to keep their databases and have their customers, their members… they don’t want to be all mixed in a huge thing and then buy back the data… All these databases have developed slowly, step by step… they don’t let them go easily. But minds change sometimes. You need time but ideas change. (DS, breeding association)

If Barto succeeds in centralising farm data at the national level, regional actors will lose the key role they play at the interface between databases and farmers. While explaining the importance of regional databases, regional administrations emphasise their knowledge of the local agricultural context and highlight their role as brokers between, on the one hand, distant and abstract bureaucratic forms and procedures, and on the other hand, the specific, localised situation of individual farmers and their practices. This confirms Eastwood et al. (2019) point about the important role played by local advisors as “sensemakers” in the development of digital technologies. A centralised system, without this level of translation, would be detrimental to the farmers who would face requests for data of which they cannot make sense, further increasing the gap between abstract policy instruments and the reality of their farms. Wittingly or unwittingly, Barto provokes competition for a gatekeeper function between farmers and databases in the digital agriculture assemblage.

In comparison to the difficult development of Barto, eFishery can be seen as a success story in the context of digitalisation projects. The cofounders of eFishery claimed that they were to a certain extent on their way to achieving their desire and dream of a more efficient and just food system. We noticed that the startup, as well as the farmers enrolled in the assemblage, realised that digitalisation had the potential of reconfiguring what for many years had been a stable, immutable market for fish commodities. The intricacies of a traditional fish supply chain—involving rent-seeking activities by the middlemen who link feed companies with farmers, and farmers with the traditional fish market in Jakarta—can be abruptly changed and disrupted by means of digital technology. Farmers are now able to benefit from the system through better access to feed supplies, markets, and a more stable commodity price.

However, the everyday digitalisation of fisheries led by eFishery is not without its critics. While most of the affiliated farmers did not see any issue with having their data used without consent by external actors (whether financial institutions, feed suppliers, or fish buyers) that seek to benefit from aggregated farm and farmer data, there are some who address this in a rather different discourse. Similar to Barto, data ownership and access issues were sometimes raised in discussions about business ethics. The eFishery manager argued that the farm data collected was actually owned by the startup; the machine collects, acquires, and compiles the raw data, the analysts translate it into meaningful information, and the farmers benefit from the overall process, making this a win–win scenario.

One Internet-of-Things (IoT) expert that we interviewed acknowledges that Indonesia’s digitalisation of agriculture was far from addressing issues of data ownership and ethical questions related to big data management—the industry is still grappling with farmers’ acceptance of, and ability to adapt to, new technology, the messiness of actors’ relations and data associated with them, and the risk that the sector poses for investments:

It’s just like the e-commerce frenzy years ago, where they started with a whole lot of small startups, but they faded away and what was left was only two or three big players. This is where investment goes. But then again, I’m not so sure about this digital agriculture. The food system in Indonesia is too complex, no agri-digital startups can last long enough in this field. It’s not attracting investment either. I’ve been in this business for years, I know… there are too many small farmers that are too old and unreceptive to new technologies. (ED, e-commerce)

This claim is substantiated by the fact that despite rapid growth, the startup business model adopted by eFishery is in itself precarious. It is subject to ongoing contestation and reconfiguration, and although visible to the public (in this case, business investors), is not necessarily robust in stature. To refer to Jasanoff (2015), what is demonstrated in the case of eFishery is the way a project is held and stabilised. In the case of eFishery, the company did not—at least not during the period of our fieldwork—show a positive cash flow; rather, it relied on grants and venture capital.

Not one business has actually shown a sustainable cash flow. How are they going to manage all those small farmers? What happens if the farmers can’t return their investment? I think they [the startups] are still relying on venture capitals to survive. (IJ, senior manager in a blockchain-based startup)

Our empirical findings show that reflexive digitalisation is not only about putting a critical lens on digitalisation. It is also a reciprocal process through which machinic relations that are built in the everyday assemblage—along with the desire imposed by a project (and actors that desire it) onto other actors—provoke the individual actor to think, do, and communicate. It is not a cyclical process by which a project leads to the everyday which then leads to reflexivity. As a multiplicity, the three emerge as one, simultaneously and reciprocally. Barto and eFishery are embracing their own projects, whilst at the same time experiencing the everyday through their engagement with the materiality of the digital, but also reflexively building a discursive process that, in the end, may or may not inform the way they shape their desire and engage with the everyday.

## Conclusion

This article has proposed a way of seeing digitalisation as an assemblage, and in doing so has identified that digitalisation manifests itself in three dimensions or facets: as project, the everyday, and reflexivity. We have shown that these three facets do not occur in isolation, but inform, provoke, and transform each other. These vibrant processes unfold as the collective enunciation intermingles with the machinic, lines of articulation, and lines of flight.

The conceptual framework developed in this article is useful for at least four reasons. Firstly, it helps differentiate between sociotechnical imaginaries and actual digitalisation processes. Too often, these two different, yet related, aspects are conflated. The conceptualisation of digital assemblages as lying at the interface of the enunciative and the machinic acknowledges the fundamental difference between projects emerging from a collective desire and their messy enactment in the everyday, as a result of a distributive form of agency. At the same time, this framing also highlights the interrelations between imaginaries and concrete networks of relations. As the Swiss case showed, the digital bureaucratisation project did not produce the administrative simplification it promised. Rather, it contributed to the current transformation of agricultural governance while at the same time being reshaped by diverse forms of resistance and unexpected outcomes.

Secondly, the framework allows for comparative approaches. Through the three facets of digitalisation, we can understand how similar imaginaries are produced and transformed in contact with diverse assemblages, or inversely, how different projects emerge from diverging enactments of similar technologies. In our case studies, the similarity between these projects of digitalisation resulted—at the very general level—form a desire to bring order into the messy world of data. However, unfolding in very different agricultural, political, and societal contexts, they resulted in very different digitalisation processes.

Thirdly, the framework directs attention to the effects of an everyday, ordinary digitalisation that is very often hidden by spotlight digitalisation. The literature on agri-food digitalisation has, in its vast majority, concentrated on highly innovative practices and digital tools (precision agriculture, robots, etc.). While this is no doubt a very important field of enquiry, it ignores and even renders invisible many other aspects central to the digitalisation of agriculture and agri-environmental governance. These include, for instance, the increased bureaucratisation of agriculture identified in our Swiss case study and the alternative uses made of these new technologies by less powerful actors (e.g. the increased use of smartphones by farmers, not necessarily for the purpose of connecting them to the advanced technologies offered by startups).

Lastly, the model acknowledges the multiple dimensions of agency: collective, distributed, and reflexive. At the same time, it emphasises the limited capacity of single actors to steer assemblages in either their projective or reflexive engagement with digitalisation. The fact that, in all of the three dimensions identified, agency is always relational calls for a reformulation of the understanding of power in assemblage thinking. Our nuanced approach to agency offers a useful starting point for rejuvenating our engagement, as social scientists, with the politics of technologies and innovation.

## Footnotes

1 The average size of the Swiss farms was 20.9 ha in 2019 (https://www.agrarbericht.ch/fr/exploitation/structures/exploitation).

2 For more details on the development of digital agri-environmental governance in Switzerland, see Forney & Epiney (forthcoming).
